# Maturation of phagosomes containing different erythrophagocytic particles in primary macrophages

**DOI:** 10.1002/2211-5463.12262

**Published:** 2017-08-23

**Authors:** Inês B. Santarino, Otília V. Vieira

**Affiliations:** ^1^ CEDOC NOVA Medical School, Faculdade de Ciências Médicas Universidade NOVA de Lisboa Portugal

**Keywords:** complement‐opsonized red blood cells, aged red blood cells, erythrophagocytosis, IgG‐opsonized Red Blood Cells, phagosomal maturation

## Abstract

Erythrophagocytosis is a physiological process that aims to remove damaged red blood cells from the circulation in order to avoid hemolysis and uncontrolled liberation of iron. Many efforts have been made to understand heme trafficking inside macrophages, but little is known about the maturation of phagosomes containing different types of erythrophagocytic particles with different signals at their surfaces. Therefore, we performed a comparative study on the maturation of phagosomes containing three different models of red blood cells (RBC): aged/senescent, complement‐opsonized, and IgG‐opsonized. We also used two types of professional phagocytes: bone marrow‐derived and peritoneal macrophages. By comparing markers from different stages of phagosomal maturation, we found that phagosomes carrying aged RBC reach lysosomes with a delay compared to those containing IgG‐ or complement‐opsonized RBC, in both types of macrophages. These findings contribute to understanding the importance of the different signals at the RBC surface in phagolysosome biogenesis, as well as in the dynamics of RBC removal.

**Abbreviations**
agRBC: aged red blood cells
BMDM: bone marrow‐derived macrophages
EP: erythrophagocytosis
PM: peritoneal macrophages
PS: phosphatidylserine
RBC: red blood cells
shRBC: sheep red blood cell

Red blood cells/erythrocytes (RBC) undergo a maturation process in which the precursor cells lose their nuclei and organelles while accumulating hemoglobin. Mature normal human RBC remain in the circulation for roughly 120 days before removal. Aged/senescent RBC are phagocytized by macrophages of the reticuloendothelial system, found in the spleen, liver, and bone marrow, through a process termed erythrophagocytosis (EP) 1. This highly controlled and coordinated process is responsible for RBC degradation within the phagolysosome resulting in the breakdown of hemoglobin and the release of heme into the cytosol where its catabolism takes place, leading to iron release 1. The rapid removal of RBC from the circulation is vital for maintaining iron/heme homeostasis, as the majority of iron required to sustain erythropoiesis is derived from senescent RBC and free iron is a strong pro‐oxidant 2.

During aging, RBC undergo several metabolic and physical modifications such as (a) membrane vesiculation, disposing nonfunctional membrane patches 3; (b) oxidation‐induced modifications of hemoglobin and membrane protein band 3 4; (c) energy depletion 5; (d) progressive cell shape transformation 6; and (e) membrane remodeling, such as the exposure of surface removal markers like phosphatidylserine (PS) 7. Directly or indirectly, these modifications trigger erythrophagocytosis.

It has been shown that the central step in the clearance of RBC relies on the interaction between macrophage receptors and the protein band 3 that upon hemoglobin oxidation undergoes clustering, promoting the generation of epitopes on the RBC surface. This provides a signal favoring recognition of redistributed band 3 by autologous IgG 8, 9. Despite this, the immunoglobulins formed are not efficient opsonins, due to their low affinity and low circulation numbers. For this reason, it has been hypothesized that phagocytosis of RBC can be enhanced by the activation of the classical complement pathway after IgG binding 10. These immunoglobulins preferentially generate C3b2–IgG complexes in the presence of active complement 11. Furthermore, prior to senescence, RBC may enter eryptosis, a form of stress‐inducing programmed cell death resembling apoptosis in nucleated cells. It is characterized by RBC shrinkage and translocation of PS from the inner leaflet to the outer leaflet of the membrane 12. Despite some *in vivo* data showing PS exposure upon RBC aging, there is no convincing evidence for the involvement of PS in the physiological removal of aged RBC 13, 14. However, this mechanism remains inadequately defined. Recently, it was shown that senescent RBC are more prone to PS exposure than young RBC upon oxidation 15.

The bulk of the iron required to synthesize new hemoglobin is derived from senescent RBC that have undergone erythrophagocytosis; it is important to understand this process better. Furthermore, in some pathologies such as chronic kidney disease and sepsis, there is a decreased RBC lifespan and there is an increased eryptosis 16, 17, making a detailed understanding of the kinetics of erythrophagocytosis under different conditions important. Thus, it is fundamental to define the mechanism of RBC clearance as well as the role of the different signals at the RBC surface, such as IgG, complement, and PS, in this process.

In this study, we aimed to address the interaction of different erythrophagosomes containing IgG‐ or complement‐opsonized RBC or PS‐enriched RBC (aged RBC) with components of the endocytic pathway, namely early endosomes and lysosomes. To do this, we used primary bone marrow‐derived mouse macrophages (BMDM) and peritoneal cavity macrophages (PM). In both BMDM and PM, phagosomes containing aged RBC mature slower than phagosomes containing the other RBC models and this information could offer an improved understanding of pathologies with changes in RBC clearance.

## Materials and methods

### Cell culture

The L929 cell line (kindly provided by Ira Tabas, Columbia University, NY, USA) was routinely cultured to produce L‐cell conditioned media (LCCM) enriched in mouse colony‐stimulating factor (M‐CSF) to differentiate monocytes into macrophages, as previously described 18.

Bone marrow‐derived macrophages (BMDM) and resident peritoneal macrophages (PM) were obtained from eight‐ to ten‐week‐old C57BL/6 wild‐type mice. BMDM were maintained for 7 days as described 19 but in RPMI‐1640 medium containing 10% FBS, 1% P/S (Gibco, Gaitherburg, MD, USA), and 30% LCCM. PM were collected from the mice peritoneal cavity and were maintained in DMEM high glucose (Gibco) supplemented with 20% FBS and 1% P/S and then plated in glass coverslips. After 2‐h incubation, nonadherent cells were removed and the adherent cells were washed with warm sterile PBS followed by replacement with fresh DMEM 19.

C57BL/6 mice were purchased from Charles River and were maintained according to the protocols approved by the national (Portuguese Official Veterinary Department; Direcção Geral de Veterinária) ethics committees according to the Portuguese (Decreto‐Lei 113/2013) and European (Directive 2010/63/EU) legislations.

### Preparation of the different phagocytic particles

Human blood was collected into sodium heparin tubes (Greiner Bio‐One, Kremsmünster, Austria) from healthy volunteers. Each volunteer signed a consent form approved by the Ethical Review Board of the NOVA Medical School, New University of Lisbon. Sheep blood was collected from healthy sheep in a slaughterhouse. All protocols were authorized by the national (Portuguese Official Veterinary Department, Direcção Geral de Veterinária) ethics committees according to Portuguese (Decreto‐Lei 113/2013) and European (Directive 2010/63/EU) legislation. Both human and sheep RBC (shRBC) were isolated using the same protocol 20. Briefly, RBCs were isolated using a Ficoll‐Paque (GE Healthcare Life Sciences, Uppsala, Sweden) gradient. After centrifugation at 400 ***g*** for 30 min at 4 °C, RBC were located at the bottom of the centrifuge tube. Then, RBC were washed twice with PBS (137 mm NaCl, 2.7 mm KCl, 1.8 mm KH_2_PO_4_, 10 mm NaHPO_4_.2H_2_O, pH 7.4) and finally resuspended in PBS supplemented with 0.1% glucose (20% V:V). RBC in suspension were kept at 4 °C and used as native RBC.

Aged RBC (agRBC) were prepared as described previously 20. Briefly, native RBC in PBS without glucose were incubated at 37 °C for 4 days.

IgG‐opsonized RBC were prepared according to the following protocol: Fresh shRBC were fixed with 4% paraformaldehyde/sucrose at room temperature (RT) for 2 h 30 min in an orbital rotator before IgG opsonization. IgG opsonization was performed overnight at 4 °C with a rabbit anti‐sheep RBC antibody (Sigma‐Aldrich, Darmstadt, Germany) at a ratio of 1 : 50.

C3bi‐opsonized shRBC were prepared according to the protocol previously described 21. Briefly, a 10% shRBC suspension was mixed with PBS and 180 ng·mL^−1^ rabbit anti‐sheep IgM (Cedarlane, Burlington, NC, USA) and incubated at RT for 1 h in an orbital rotator. Next, IgM‐shRBC were incubated with C5‐deficient human serum (Sigma‐Aldrich) at a ratio of 1 : 10 (v/v). The mixture was incubated for 20 min at 37 °C in a thermomixer at 300 rpm. Under these conditions, the Fc region of the IgM pentamer activates the classical pathway of the complement cascade and deposits C3b to the IgM‐shRBC where it is rapidly converted to C3bi.

Before phagocytosis and to allow visualization under the microscope, agRBC, IgG‐opsonized RBC, and C3bi‐RBC were labeled with the vital dye carboxyfluorescein succinimidyl ester (CFSE) according to the CellTracer™ CFSE Cell Proliferation Kit instructions. With IgG‐opsonized RBC, these particles were incubated with CFSE prior to fixation with PFA.

### Phagocytosis and phagosomal maturation assays

Before internalization of the different RBC models, phagocytic cells were incubated at 4 °C for 7 min with agRBC or IgG‐opsonized particles and at 37 °C for 20 min with C3bi‐opsonized particles. This procedure is important because it allows different types of erythrophagocytic particles to bind to receptors on the plasma membrane of phagocytes. Furthermore, it allows the synchronization of the phagocytic process, increasing the number of phagosomes formed during the pulse (engulfment) and then a more accurate analysis of phagolysosome formation. Next, macrophages incubated with agRBC and IgG‐opsonized particles were transferred to 37 °C for engulfment to occur. For complement‐opsonized RBC, phorbol 12‐myristate 13‐acetate at a final concentration of 150 ng·mL^−1^ was added to allow engulfment.

Phagocytosis was performed during 15‐ and 20‐min incubations (pulse time, 0‐min chase time) for BMDM and PM, respectively. The duration of phagocytosis in the two types of macrophages is different because the phagocytic capacity of BMDM is higher than that of PM 22. In all phagocytosis assays, four RBC were added per macrophage. After phagocytosis, noninternalized RBC were removed by extensively washing or by hemolysis with deionized water. Then, the cells were incubated at 37 °C for the time points referenced in the graphs abscissa (chase), to follow phagosomal maturation. Next, phagosomal maturation was assessed by following the interaction of the phagosomes formed only during the pulse (0‐min chase time) with components of the endocytic pathway at different time points (chase times).

### Immunofluorescence and microscopy

After the pulse‐chase experiments, cells were fixed with 4% PFA for 30 min at RT. Early endosome antigen 1 (EEA‐1) staining was performed with mouse anti‐EEA‐1 antibody (Sigma‐Aldrich). The antibody was used at 1 : 50 dilution for 1 h at RT in cells permeabilized with 0.1% Triton X‐100/200 mm glycine in PBS for 30 min. Lysosomal‐associated membrane protein 1 (LAMP‐1) was visualized with a rat anti‐LAMP‐1 antibody (Developmental Studies Hybridoma Bank, USA). The antibody was used at 1 : 50 dilution for 2 h at RT, in cells fixed and permeabilized with methanol for 10 min. In both cases, after permeabilization and washing with PBS, blocking was performed with gelatin from cold water fish (Sigma‐Aldrich) for 30 min followed by incubation with the respective primary antibodies. Antibody‐bound cells were finally incubated with Cy3 anti‐mouse or anti‐rat secondary antibodies for 1 h at RT at 1 : 500 dilution (Jackson ImmunoResearch, West Grove, PA, USA).

Stained samples were mounted with Mowiol/DABCO (Calbiochem, Darmstadt, Germany) and analyzed by using a laser scanning confocal microscope (Carl Zeiss, Jena, Germany, lsm 510 software) with a 63x oil immersion objective N/A = 1.30. Digital images were analyzed by lsm image browser or image‐j software. A phagosome was considered positive for a given marker when a fluorescent ring or a dotted patch was observed around the engulfed particle.

### Statistical analysis

Statistical analysis (two‐way ANOVA followed by Bonferroni post‐test) was performed using the graphpad prism software version (GraphPad Software, Inc., La Jolla, San Diego, CA, USA). 5.0. *P* < 0.05 (*) and *P* < 0.001 (***) were considered to be statistically significant.

## Results and Discussion

In this study, human and sheep RBCs were used to create different models of nonopsonized and opsonized RBC. agRBC were used as a model of senescent/eryptotic cells for which the main signal for removal by primary phagocytes is the exposure of PS, as described before 20. RBC were also opsonized with C3bi to mimic complement‐mediated erythrophagocytosis and IgG to mimic Fc‐mediated erythrophagocytosis, both opsonins being involved in the *in vivo* removal of senescent erythrocytes 23, 24, 25. Because continuous subculture of cell lines may cause gene loss and impair macrophage immune function, we decided to use primary mouse macrophages as phagocytic cells*. In vivo*, the removal of senescent RBC is carried out by the macrophages of the reticuloendothelial system 26, 27, 28, 29. Splenic macrophages are considered the most active erythrophagocytes in RBC recycling, despite some evidence that the liver is the main organ responsible for EP under pathophysiological conditions 30. Due to these discrepancies and the low yield obtained from splenic macrophage isolation procedures, the majority of *in vitro* studies of erythrophagocytosis have been performed in primary cultures of mouse BMDM and PM 31, 32, 33, 34, 35, 36. Despite the fact that PM do not have a physiological role in the removal of senescent/damaged RBC, these phagocytic cells are among the best studied macrophage populations in terms of cell biology and also in the identification of the molecular mechanisms involved in the removal of senescent RBC *in vitro* 31, 33, 37. Furthermore, in this work, PM were also used as a model of resident macrophages. The peritoneal cavity has two PM subsets: large peritoneal macrophages and small peritoneal macrophages (inflammatory monocyte population) 38. In our experimental conditions, the PM population was composed mainly of large PM as the experiments were not performed in elicited peritoneal macrophages.

Erythrophagocytosis is one of the main physiological processes responsible for the maintenance of iron homeostasis. In this process, after recognition by specific receptors on the macrophage membrane surface, senescent RBC are internalized leading to the formation of a membrane‐bound vacuole termed the erythrophagosome. It resembles the plasma membrane in terms of lipid and protein composition and its fluid‐phase contents are a reflection of the extracellular milieu. These nascent phagosomes are not able to degrade cargo. However, soon after vacuole scission, there are a series of biochemical modifications that convert the nascent phagosome into a degradative organelle through an ordered choreographed succession of events. These include membrane fission and fusion of the nascent phagosomes with components of the endocytic pathway—a process known as phagosome maturation 39, 40, 41.

**Figure 1:**
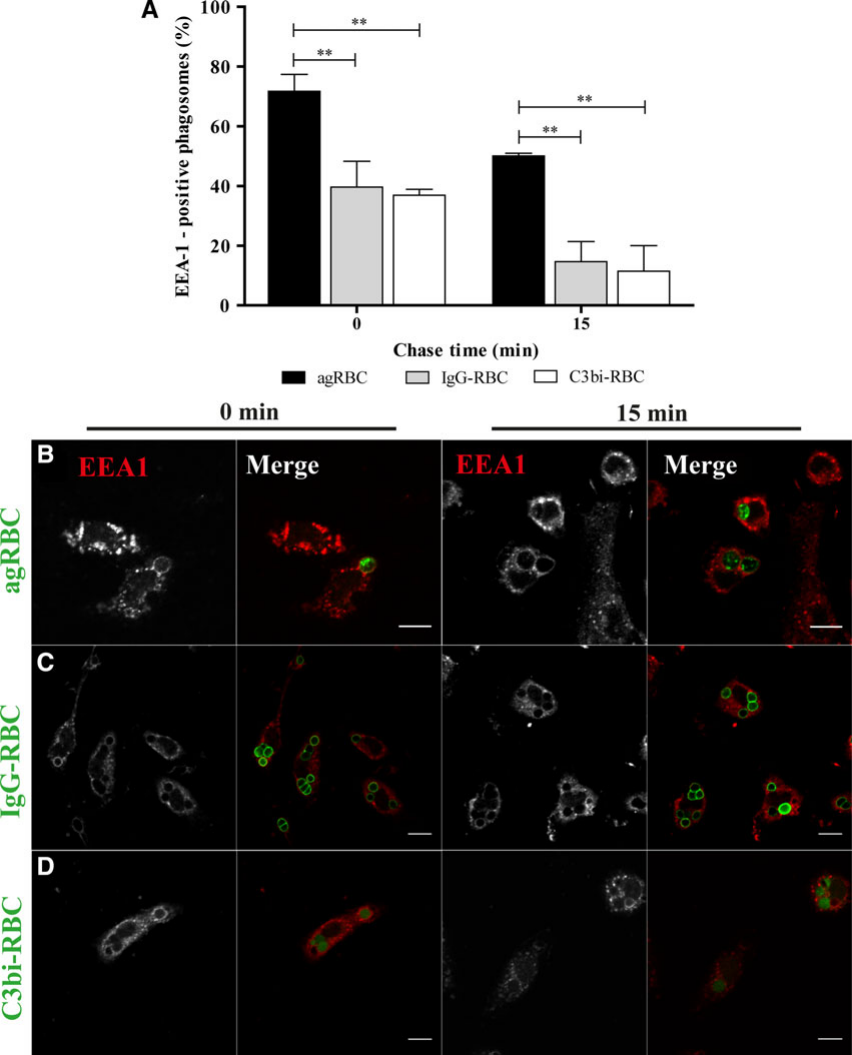
Early endosome antigen 1 acquisition by phagosomal membranes containing different phagocytic particles in bone marrow‐derived macrophages. The interaction of early endosomes with phagosomes containing different particles was assessed by the acquisition of EEA‐1 (A–D). BMDM were exposed to different phagocytic particles for 15 min and then chased for the times indicated in the graph abscissa. After pulse‐chase experiments, cells were fixed and stained with EEA‐1 antibody and the positive phagosomes for the different chase time points were quantified in images acquired under a confocal microscope. (A) Quantification of the EEA‐1‐positive phagosomes. (B) EEA‐1 staining of BMDM with agRBC‐containing phagosomes. (C) EEA‐1 staining of BMDM with IgG‐RBC‐containing phagosomes. (D) EEA‐1 staining of BMDM with C3bi‐RBC‐containing phagosomes. In B–D, for each time point, first column shows EEA‐1 distribution and the second column is a composite of EEA‐1 staining and the different RBC particles (green color). In A, the values are means ± SEM of, at least, three independent experiments. At each time point, 100 phagosomes were analyzed. **P < 0.01. Bars, 10 μm.

To assess the initial steps of phagosomal maturation, specifically the interaction between the nascent phagosome and the early components of the endocytic pathway, we followed the recruitment of the Rab5‐effector, EEA‐1 (early endosome antigen‐1), by immunofluorescence for all the phagocytic particles in BMDM. EEA‐1 is responsible for tethering early endosomes to nascent phagosomes. As observed in Fig. 1A, there was a decrease in EEA‐1 association with all the RBC‐containing phagosomes with time, suggesting that this Rab5 effector interacts transiently with the phagosomal membranes. The loss of EEA‐1 in phagosomes containing opsonized RBC was faster than in phagosomes containing agRBC. At 15‐min chase, a higher percentage of EEA‐1‐positive phagosomes was observed for phagosomes containing agRBC (50.07 ± 0.92%) *versus* (14.67 ± 6.70%) and (11.47 ± 8.57%) for IgG‐RBC and C3bi‐RBC, respectively (Fig. 1A and visualized in Fig. 1B–D).

**Figure 2:**
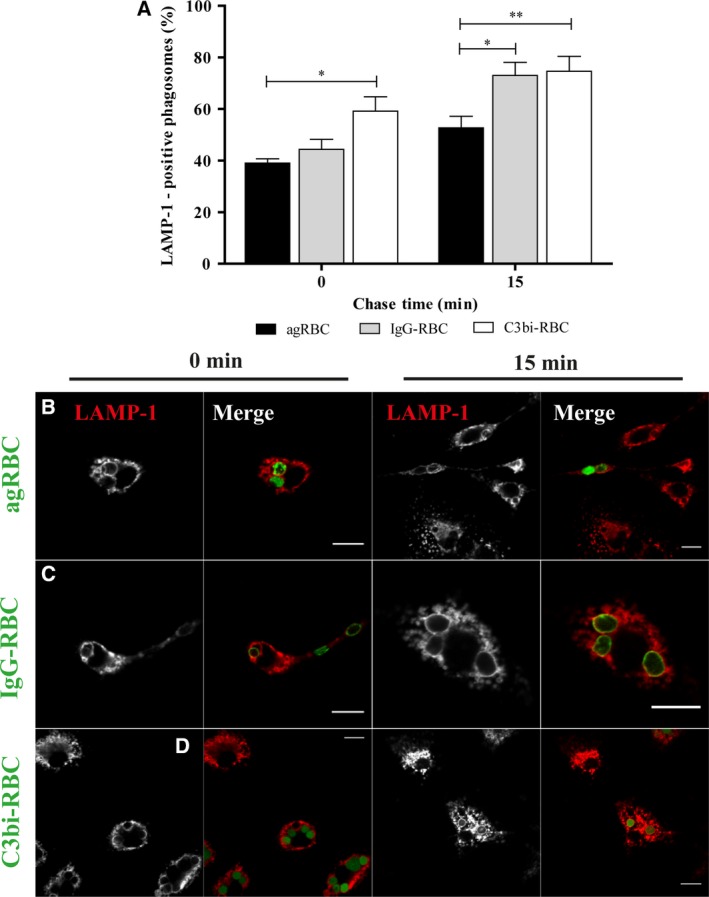
Lysosomal‐associated membrane protein 1 acquisition by phagosomal membranes containing different phagocytic particles in bone marrow‐derived macrophages. The interaction of the different phagosomes with late endocytic compartments (mainly late endosomes and lysosomes) was assessed by the acquisition of LAMP‐1 (A–D). BMDM were exposed to different phagocytic particles for 15 min and then chased for the times indicated in the graph abscissa. After pulse‐chase experiments, cells were fixed and stained with LAMP‐1 antibody and the positive phagosomes for the different chase time points were quantified in images acquired under a confocal microscope. (A) Quantification of the LAMP‐1‐positive phagosomes. (B) LAMP‐1 staining of BMDM with agRBC‐containing phagosomes. (C) LAMP‐1 staining of BMDM with IgG‐RBC‐containing phagosomes. (D) LAMP‐1 staining of BMDM with C3bi‐RBC‐containing phagosomes. In B–D, for each time point, first column shows LAMP‐1 distribution and the second column is a composite of LAMP‐1 staining and the different RBC particles (green color). In A, the values are means ± SEM of, at least, three independent experiments. At each time point, 100 phagosomes were analyzed. *P < 0.05; **P < 0.01. Bars, 10 μm.

As the phagosomes mature, interaction with late components of the endocytic pathway such as late endosomes and lysosomes occurs, culminating in erythrophagolysosome formation. RBC degradation takes place within this organelle, with subsequent trafficking of heme to the cytosol. Interaction of phagosomes containing the different RBC models with the late endocytic compartment components was assessed by the acquisition of lysosomal‐associated membrane protein (LAMP‐1) in BMDM. At 15‐min chase, LAMP‐1 was present at higher levels in IgG‐containing phagosomes (73.03 ± 5.08%) and in C3bi‐containing phagosomes (74.60 ± 5.82%), compared to agRBC‐containing phagosomes (52.70 ± 4.45%), as shown graphically (Fig. 2A) and illustrated in Fig. 2B–D. Significant differences were also observed between agRBC‐ and C3bi‐containing phagosomes (39.03 ± 1.69% and 59.20 ± 5.57%, respectively) for the same time point. These observations demonstrate that LAMP‐1 acquisition was faster in IgG‐ and C3bi‐containing phagosomes and was well correlated with the fast loss of EEA‐1 in these phagosomes (Fig. 1A).

**Figure 3:**
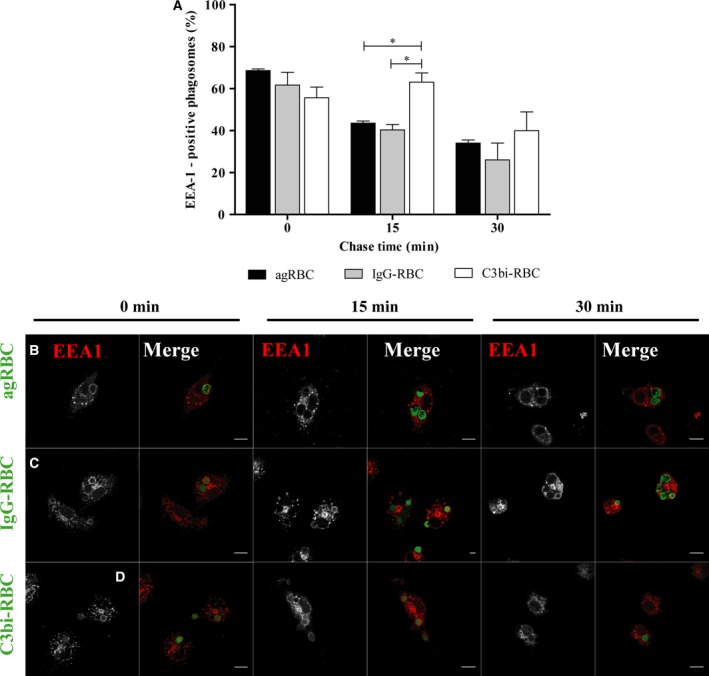
Early endosome antigen 1 acquisition by phagosomal membranes containing different phagocytic particles in peritoneal macrophages. The interaction of early endosomes with phagosomes containing different particles was assessed as described in the legend of Fig. 1. PM were exposed to different phagocytic particles for 20 min and then chased for the times indicated in the graph abscissa. After pulse‐chase experiments, cells were fixed and stained with EEA‐1 antibody and the positive phagosomes for the chase time points were quantified in images acquired under a confocal microscope. (A) Quantification of the EEA‐1‐positive phagosomes. (B) EEA‐1 staining of PM with agRBC‐containing phagosomes. (C) EEA‐1 staining of PM with IgG‐RBC‐containing phagosomes. (D) EEA‐1 staining of PM with C3bi‐RBC‐containing phagosomes. In B–D, for each time point, the first column shows EEA‐1 distribution and the second column is a composite of EEA‐1 staining and the different RBC particles (green color). In A, the values are means ± SEM of, at least, three independent experiments. At each time point, 100 phagosomes were analyzed. *P < 0.05. Bars, 10 μm.

**Figure 4:**
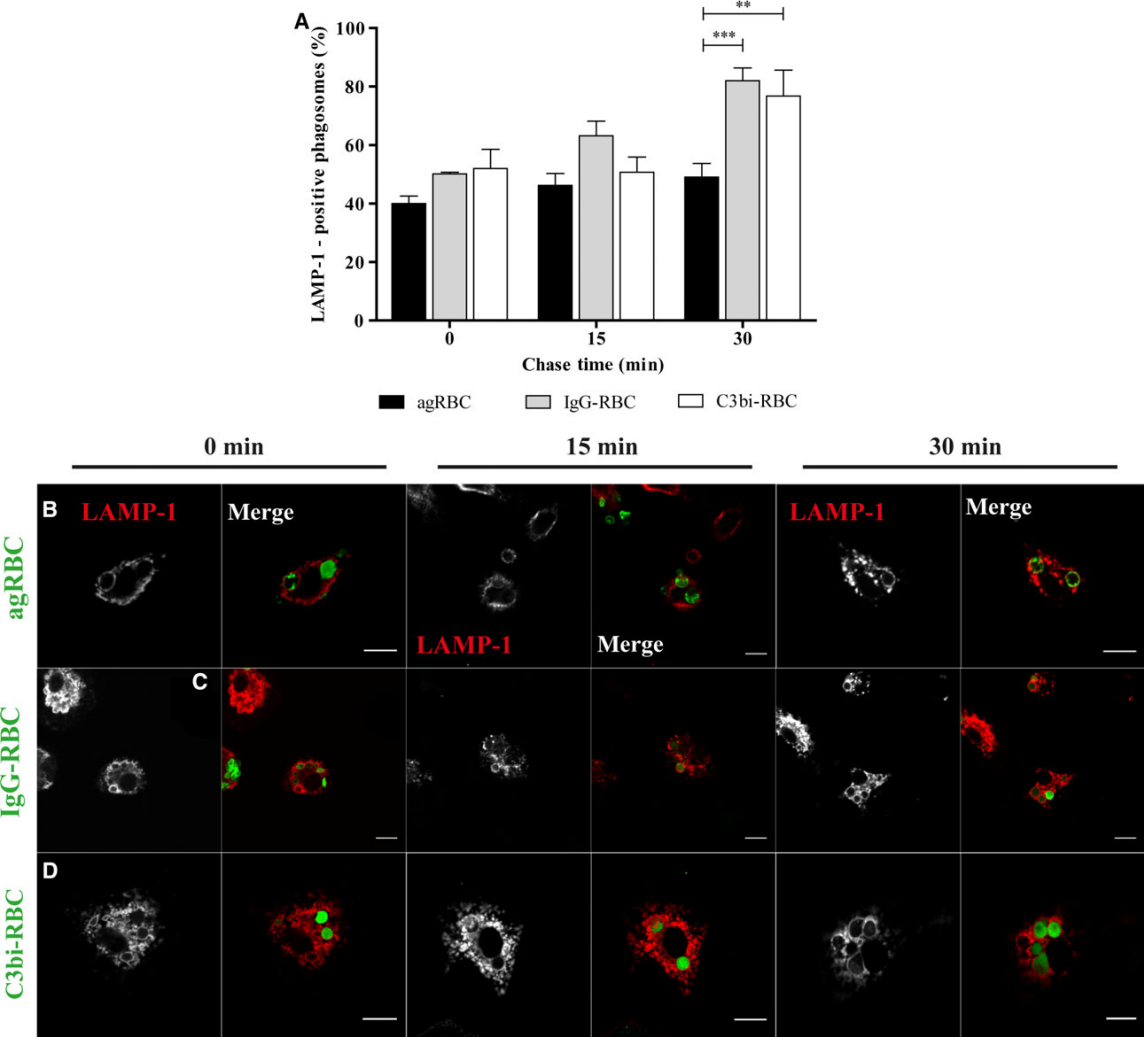
Lysosomal‐associated membrane protein 1 acquisition by phagosomal membranes containing different phagocytic particles in peritoneal macrophages. The interaction of late endosomes/lysosomes with phagosomes containing different particles was assessed as described in the legend of Fig. 2. PM were exposed to different phagocytic particles for 20 min and then chased for the times indicated in the graph abscissa. After pulse‐chase experiments, cells were fixed and stained with LAMP‐1 antibody and the positive phagosomes for the chase time points were quantified in images acquired under a confocal microscope. (A) Quantification of the LAMP‐1‐positive phagosomes. (B) LAMP‐1 staining of PM with agRBC‐containing phagosomes. (C) EEA‐1 staining of PM with IgG‐RBC‐containing phagosomes. (D) LAMP‐1 staining of PM with C3bi‐RBC‐containing phagosome. In B–D, for each time point, first column shows LAMP‐1 distribution and the second column is a composite of LAMP‐1 staining and the different RBC particles (green color). In A, the values are means ± SEM of, at least, three independent experiments. At each time point, 100 phagosomes were analyzed. **P < 0.01; ***P < 0.001. Bars, 10 μm.

The kinetic profile for maturation of phagosomes containing different RBC in PM was followed as described above for BMDM. Again, the association of EEA‐1 with phagosomal membranes was transient for the three different phagocytic particles (Fig. 3A). However, EEA‐1 remained associated with phagosomes containing C3bi‐opsonized RBC for longer periods of time compared with the other phagosomes and a significant statistical difference was observed for 15‐min chase. Indeed, at 15‐min chase time (63.10 ± 4.33%), the phagosomes containing C3bi‐opsonized RBC were still positive for EEA‐1 in contrast with (43.5 ± 1.03%) and (40.33 ± 2.52%) for agRBC‐ and IgG‐containing phagosomes, respectively (quantified in Fig. 3A and visualized in Fig. 3B–D). However, the delay in losing EEA‐1 by the phagosomes containing C3bi‐opsonized RBC did not have major consequences for the interaction of these phagosomes with late endocytic components (Fig. 4A–D). Similar to what was observed for BMDM, phagolysosome formation was faster for opsonized RBC‐containing phagosomes than for those containing agRBC.

Comparing the results obtained in Figs 2 and 4, phagolysosome biogenesis is slower in PM than in BMDM for all RBC particles. This outcome can explain, at least in part, the lower phagocytic capacity of PM in comparison with BMDM as previously reported 42. Indeed, during erythrophagolysosome biogenesis, besides membrane fusion with components of the endocytic pathway, membrane fission also occurs. This latter process is critical for recycling some phagosomal membrane components, such as phagocytic receptors, back to the macrophage plasma membrane where they will participate in the engulfment of new RBC. Thus, a delay in phagolysosome biogenesis will affect phagocytosis.

In addition to their differing origins 38, 43, BMDM and PM also have different gene expression profiles for the type and abundance of phagocytic receptors at their cell surfaces 44. This might influence the homeostatic turnover of the different RBC models. For instance, BMDM express more high‐affinity IgG binding receptors than PM and the profile of the receptors that can be involved in the internalization of aged RBC is completely different 44.

Our results also showed that in both types of primary macrophages, phagolysosome formation is faster for opsonized RBC‐containing phagosomes than for those containing agRBC. These results are similar to those obtained by our group for smooth muscle cells 20, suggesting that RBC processing is conserved among professional and nonprofessional phagocytes. Although both types of macrophages have several PS receptors on their surface that could recognize agRBC, the PS receptor TIM‐4 seems to be critical in the engulfment process for both type of macrophages. TIM‐4 is highly expressed on PM 38 and is also the main receptor mediating phagocytosis of PS‐bearing targets in BMDM 45. This receptor is involved in phagosome stabilization via actin polymerization 46, which may explain why phagolysosome biogenesis of agRBC particles is slower than phagolysosome biogenesis of opsonized RBC. Indeed, the interaction of the newly formed phagosomes with components of the endocytic pathway only occurs after actin depolymerization on the nascent phagosome. Therefore, inhibition or a delay in actin depolymerization around phagosomes will have a negative impact on phagolysosome biogenesis. Furthermore, and in contrast with Fc‐ and complement‐mediated phagocytosis, PS receptor‐mediated phagocytosis is immunologically silent and induces an anti‐inflammatory response 47. This can also explain why phagolysosome biogenesis of agRBC is slower than opsonized RBC phagolysosome biogenesis. Indeed, as Fc and complement receptors are involved in the rapid removal and killing of bacteria from the circulation to avoid infection, it is to be expected that phagosomes whose formation depends on these receptors have to mature rapidly. Additionally, phagocytosis through either of these receptors usually results in the formation of reactive oxygen species and pro‐inflammatory cytokine secretion 48, 49. Finally, our data also demonstrate that different opsonins that interact with different phagocytic receptors leading to different signaling responses do not result in major differences in terms of phagolysosome biogenesis in both types of primary macrophages.

In conclusion, this work identifies the differences involved in erythrophagocytosis of different *in vitro* RBC models in two different types of macrophages. Although the phagocytic cells and phagocytic particles of different origin could limit to some extent the conclusions at the physiological level, our findings are a step forward in the identification of the molecular mechanisms of agRBC engulfment, which remain poorly understood.

## Author contributions

OVV conceived and designed the project. IBS acquired the data. OVV and IBS analyzed and interpreted the results. OVV and IBS wrote the manuscript.
